# Reduced activity in a liaison psychiatry service during the peak of the COVID-19 pandemic: Comparison with 2019 data and characterisation of the SARS-COV-2 positive cohort

**DOI:** 10.1192/j.eurpsy.2021.735

**Published:** 2021-08-13

**Authors:** M. Butler, A. Delvi, F. Mujic, S. Broad, L. Pauli, T. Pollak, S. Gibbs, C.C. Sin Fai Lam, M. Calcia, S. Posporelis

**Affiliations:** 1 Psychological Medicine, South London and Maudsley NHS Foundation Trust, London, United Kingdom; 2 Psychosis Studies, Institute of Psychiatry, Psychology and Neuroscience, London, United Kingdom; 3 Faculty Of Life Sciences And Medicine, King’s College London, London, United Kingdom; 4 Section Of Women’s Mental Health, Institute of Psychiatry, Psychology and Neuroscience, London, United Kingdom

**Keywords:** liaison psychiatry, Covid, pandemic

## Abstract

**Introduction:**

The COVID-19 pandemic led to changes in how healthcare was accessed and delivered. It was suggested that COVID-19 will lead to an increased delirium burden in its acute phase, with variable effect on mental health in the longer term. Despite this, there are limited data on the direct effects of the pandemic on psychiatric care.

**Objectives:**

1) describe the mental health presentations of a diverse acute inpatient population, 2) compare findings with the same period in 2019, 3) characterise the SARS-CoV-2 positive cohort of patients.

**Methods:**

We present a descriptive summary of the referrals to a UK psychiatric liaison department during the exponential phase of the pandemic, and compare this to the same period in 2019.

**Results:**

show a 40.3% reduction in the number of referrals in 2020, with an increase in the proportion of referrals for delirium and psychosis. One third (28%) of referred patients tested positive for COVID-19 during their admission, with 39.7% of these presenting with delirium as a consequence of their COVID-19 illness. Our data indicate decreased clinical activity for our service during the pandemic’s peak. There was a marked increase in delirium, though in no other psychiatric presentations.

**Conclusions:**

In preparation for further exponential rises in COVID-19 cases, we would expect seamless integration of liaison psychiatry teams in general hospital wards to optimise delirium management in patients with COVID-19. Further consideration should be given to adequate staffing of community and crisis mental health teams to safely manage the potentially increasing number of people reluctant to visit the emergency department.

